# Supplementary material for: Slow recombination of spontaneously dissociated organic fluorophore excitons
Source: Nat Commun. 2019 Dec 17;10:5748. doi: 10.1038/s41467-019-13736-8 (PMC6917757; doi:10.1038/s41467-019-13736-8)
Supplement: Supplementary file 1 — Supplementary Information [file 41467_2019_13736_MOESM1_ESM.pdf]

Supplementary Information:

Slow recombination of spontaneously dissociated organic fluorophore excitons

Takahiko Yamanaka[1,2], Hajime Nakanotani[2,3], and Chihaya Adachi[2,3,]

1. Central Research Laboratory, Hamamatsu Photonics K.K., 5000 Hirakuchi, Hamakita-ku, Hamamatsu, Shizuoka 434-8601, Japan
2. Center for Organic Photonics and Electronics Research (OPERA), Kyushu University, 744 Motooka, Nishi-ku, Fukuoka 819-0395, Japan
3. International Institute for Carbon-Neutral Energy Research (WPI-I2CNER), Kyushu University, 744 Motooka, Nishi-ku, Fukuoka 819-0395, Japan

Correspondence and requests for materials should be addressed to T.Y. (takahiko.yamanaka@crl.hpk.co.jp), H.N. (nakanotani@cstf.kyushu-u.ac.jp), C.A. (adachi@opera.kyushu-u.ac.jp).

Contents:

Supplementary Figures: 1-10

Supplementary Figures

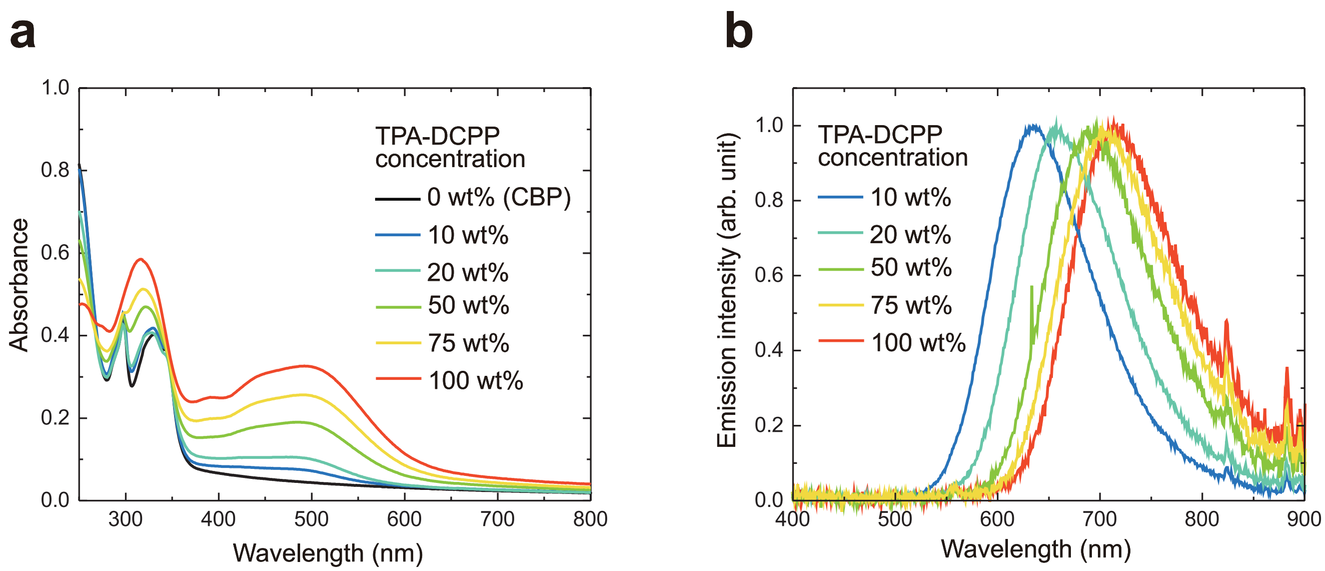

Supplementary Figure 1. PL properties of TPA-DCPP-doped solid-state films. a, Absorption spectra of TPA-DCPP:CBP blends with different TPA-DCPP doping concentrations. b, Fluorescence spectra of TPA-DCPP:CBP blends with different TPA-DCPP doping concentrations.

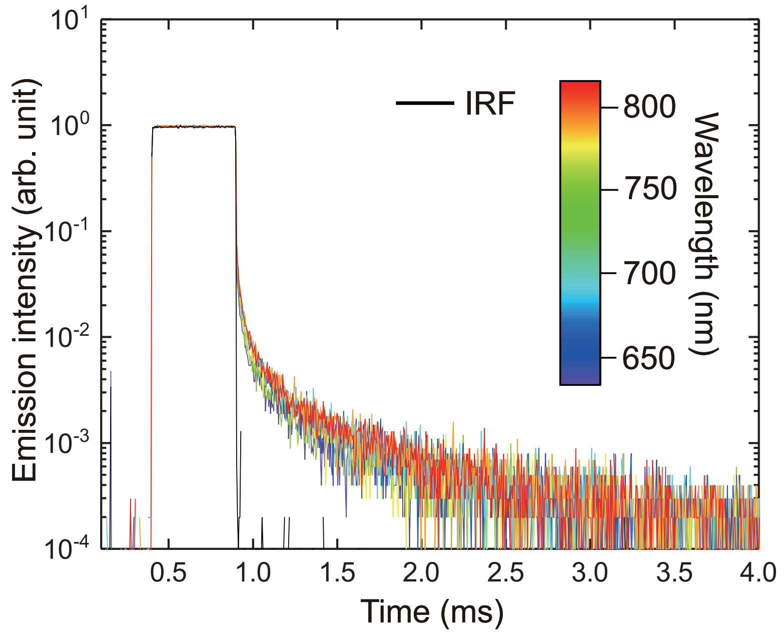

Supplementary Figure 2. Dependence of TRPL profile on detection wavelength in a 50-wt%-TPA-DCPP:CBP blend. The black line indicates the IRF of the fluorescence lifetime spectrometer. A 470-nm light source was used as excitation light, and the power density was fixed at 25 mW cm^{-2}.

Supplementary Figures

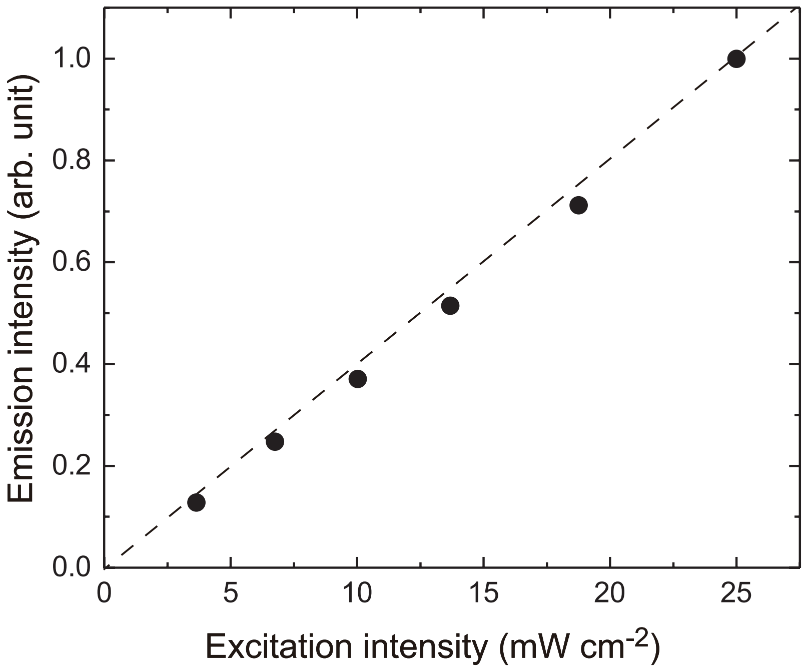

Supplementary Figure 3. Dependence of emission intensity on excitation light power in a 50-wt%-TPA-DCPP:CBP blend.

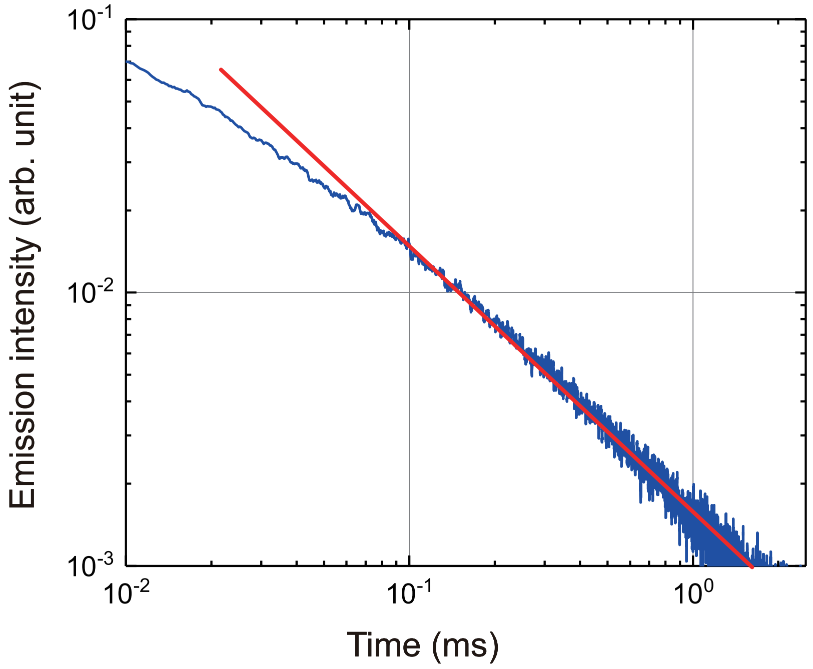

Supplementary Figure 4. Log-log plot of the TRPL profile (excitation pulse width = 500 μs) of a 50-wt%-TPA-DCPP:CBP blend. The red line indicates a power-law kinetic, *i.e.*, emission intensity = t^{-m} (m = 1).

Supplementary Figures

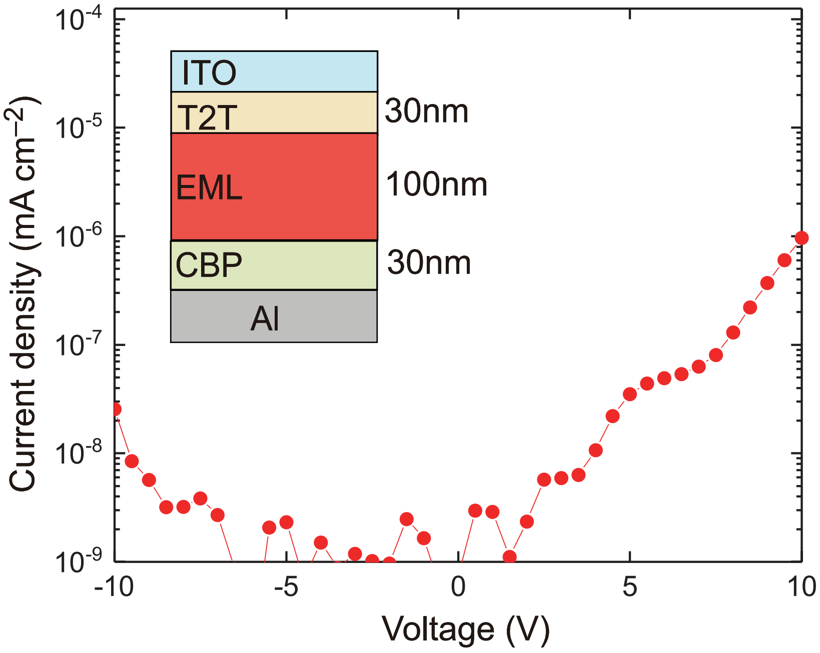

Supplementary Figure 5. Current density-voltage characteristics of a non-injecting device. The current density is below the nA cm^{-2} level even at 5 V, confirming no carrier injection. Inset: the device structure.

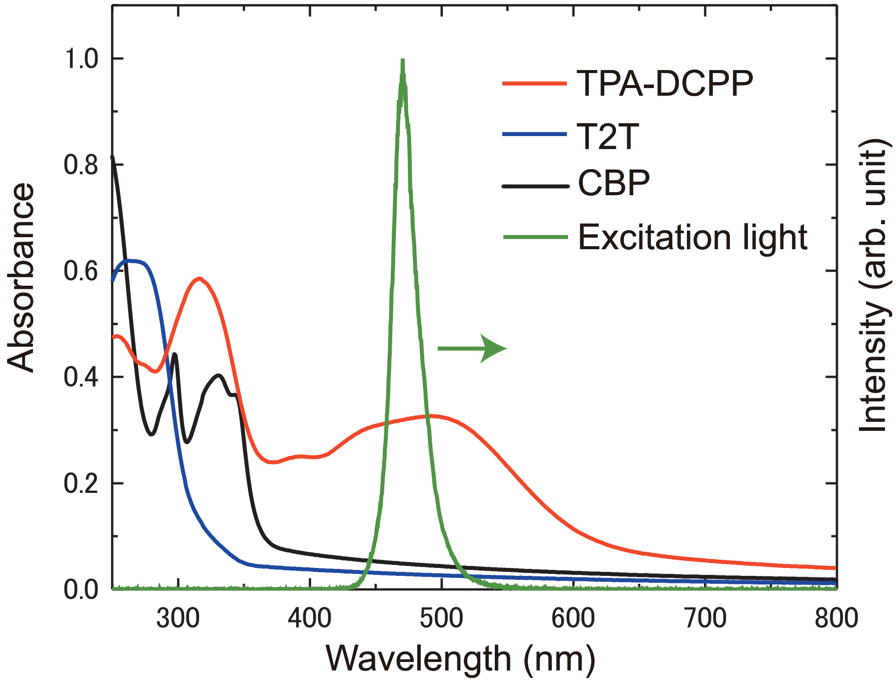

Supplementary Figure 6. Absorption spectra of CBP, T2T, and TPA-DCPP films. The green line indicates the spectrum of excitation light.

Supplementary Figures

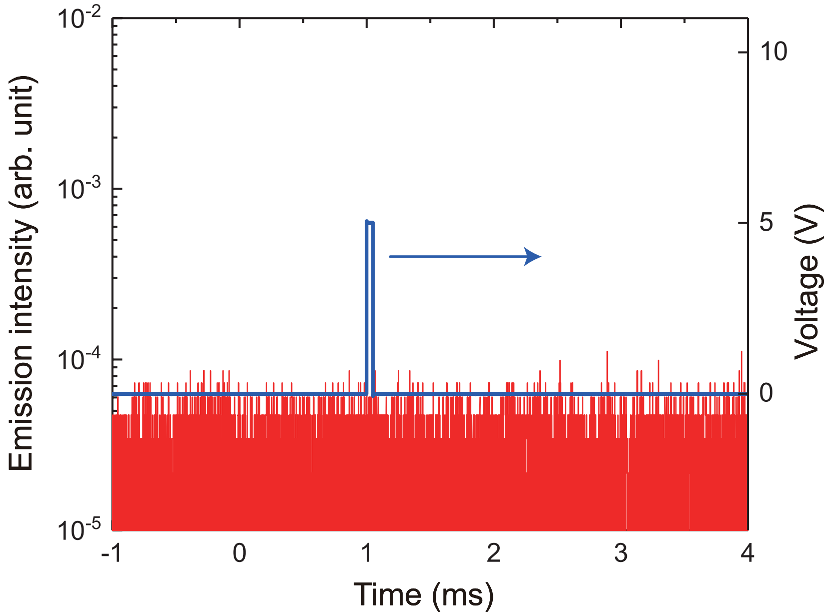

Supplementary Figure 7. TRPL profile (red line) in a 50-wt%-TPA-DCPP:CBP non-injecting device (the same device used in the measurement shown in Fig. 2) that was not photo-excited before applying the voltage. The blue line indicates the time profile of the external applied voltage.

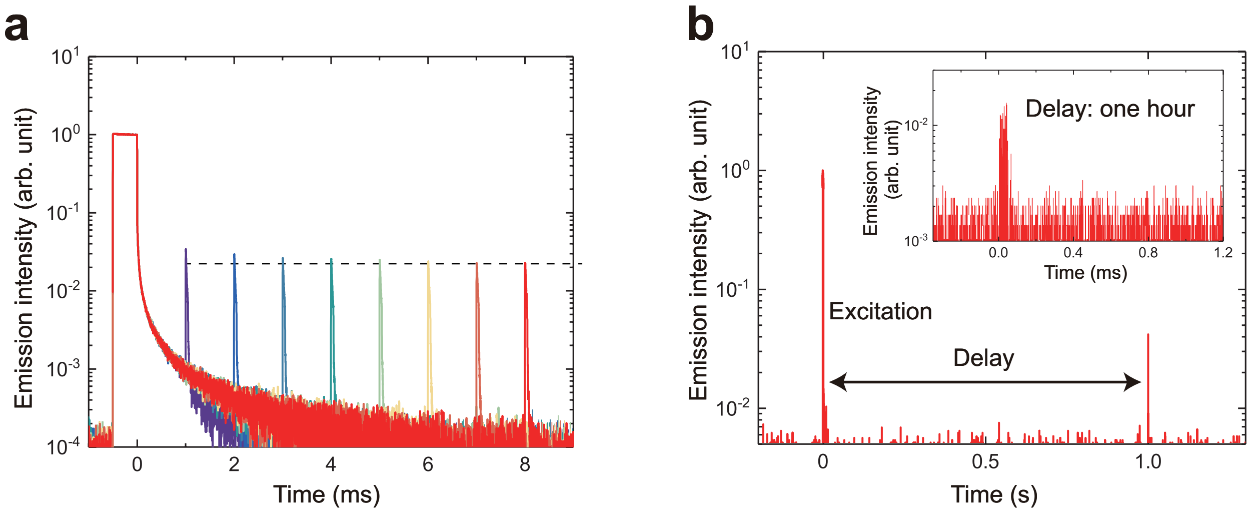

Supplementary Figure 8. Dependence of the external-electric-field-modulated TRPL profiles on external-voltage-pulse delay (**a**: 10 ms range, **b**: second range, **b inset**: hour range) in the 50-wt%-TPA-DCPP:CBP non-injecting device. The excitation light pulse width was set to 500 μs. The applied voltage and width were fixed to 5 V and 50 μs, respectively. The TRPL signals for the one-hour-delay measurement were averaged just 10 times because of equipment limitations.

Supplementary Figures

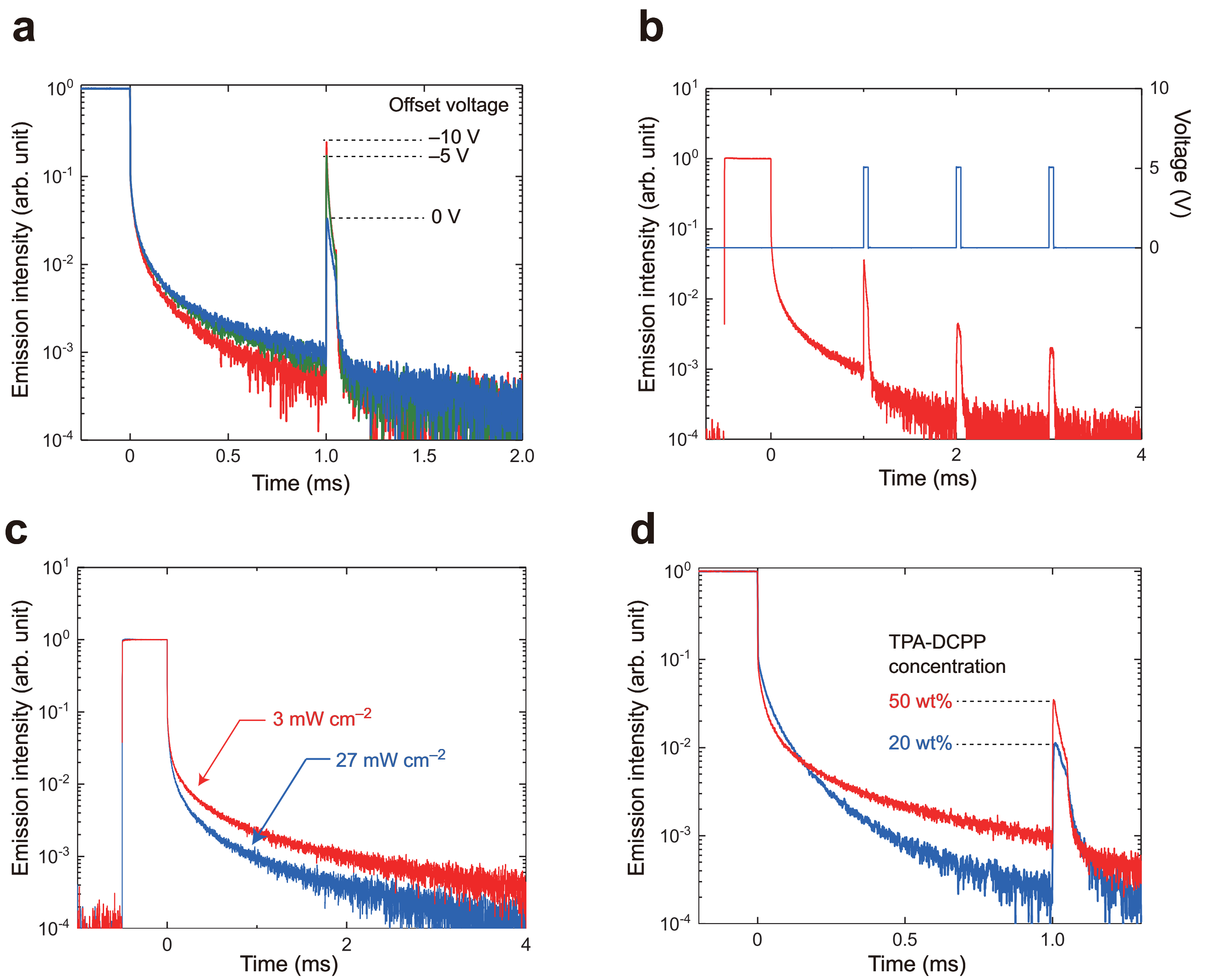

Supplementary Figure 9. a, Dependence of the emission spike intensity in TRPL profiles on the offset voltage for the 50-wt%-TPA-DCPP:CBP non-injecting device. The dashed lines indicate the maximum intensity for each condition. Note that the prompt decay profiles (from 0 to 1 ms) also show an offset-voltage dependence. **b**, External-electric-field-modulated TRPL profiles (red line) with multiple voltage pulses (blue line) for the 50-wt%-TPA-DCPP:CBP non-injecting device. The applied voltage and width were fixed at 5 V and 50 μs, respectively. **c**, Dependence of the TRPL decay profile on the excitation light power (3 or 27 mW cm^{-2}) for a 50-wt%-TPA-DCPP:CBP blend. **d**, Dependence of the emission spike intensity in the TRPL profile on the doping concentration for TPA-DCPP:CBP non-injecting devices. The dashed lines indicate the maximum intensity for each concentration. Note that the prompt decay profiles (from 0 to 1 ms) also show a concentration dependence.

Supplementary Figures

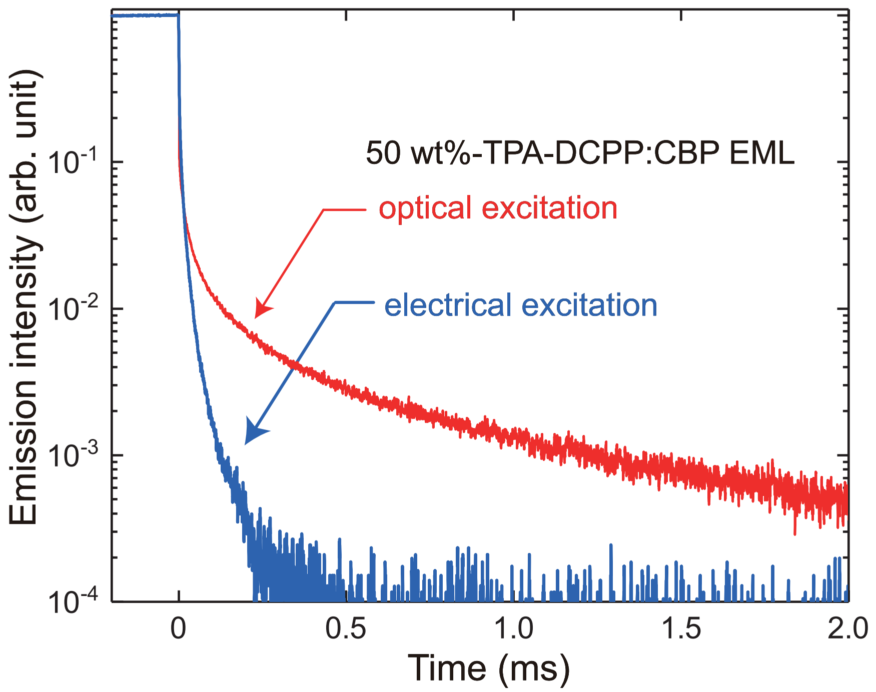

Supplementary Figure 10. Comparison of transient emission decay profiles under optical and electrical excitation. The device structure was ITO (100 nm) / TAPC (50 nm) / EML (30 nm) / T2T (20 nm) / BPy-TP2 (60 nm) / LiF (1.6 nm) / Al (100 nm). For electrical excitation, a constant current density (1.0 mA cm^{-2}) was injected into the device for 500 µs.
